# Long noncoding RNA SNHG14 promotes hepatocellular carcinoma progression by regulating miR-876-5p/SSR2 axis

**DOI:** 10.1186/s13046-021-01838-5

**Published:** 2021-01-23

**Authors:** Zhibin Liao, Hongwei Zhang, Chen Su, Furong Liu, Yachong Liu, Jia Song, He Zhu, Yawei Fan, Xuewu Zhang, Wei Dong, Xiaoping Chen, Huifang Liang, Bixiang Zhang

**Affiliations:** 1 Hepatic Surgery Center, Tongji Hospital, Tongji Medical College, Huazhong University of Science and Technology, Wuhan, Hubei P. R. China; 2 Hubei Key Laboratory of Hepato-Pancreato-Biliary Diseases, Wuhan, Hubei P. R. China; 3 Key Laboratory of Organ Transplantation, Ministry of Education, Wuhan, Hubei P. R. China; 4 Key Laboratory of Organ Transplantation, Chinese Academy of Medical Sciences, Wuhan, Hubei P. R. China

**Keywords:** SNHG14, Hepatocellular carcinoma, miR-876-5p, SSR2, ceRNA

## Abstract

**Background:**

Aberrant expressions of long noncoding RNAs (lncRNAs) have been demonstrated to be related to the progress of HCC. The mechanisms that SNHG14 has participated in the development of HCC are obscure.

**Methods:**

Quantitative real-time PCR (qRT-PCR) was used to measure the lncRNA, microRNA and mRNA expression level. Cell migration, invasion and proliferation ability were evaluated by transwell and CCK8 assays. The ceRNA regulatory mechanism of SNHG14 was evaluated by RNA immunoprecipitation (RIP) and dual luciferase reporter assay. Tumorigenesis mouse model was used to explore the roles of miR-876-5p in vivo. The protein levels of SSR2 were measured by western blot assay.

**Results:**

In this study, we demonstrated that SNHG14 was highly expressed in HCC tissues, meanwhile, the elevated expression of SNHG14 predicted poor prognosis in patients with HCC. SNHG14 promoted proliferation and metastasis of HCC cells. We further revealed that SNHG14 functioned as a competing endogenous RNA (ceRNA) for miR-876-5p and that SSR2 was a downstream target of miR-876-5p in HCC. Transwell, CCK8 and animal experiments exhibited miR-876-5p inhibited HCC progression in vitro and in vivo. By conducting rescue experiments, we found the overexpression of SSR2 or knocking down the level of miR-876-5p could reverse the suppressive roles of SNHG14 depletion in HCC.

**Conclusion:**

SNHG14 promotes HCC progress by acting as a sponge of miR-876-5p to regulate the expression of SSR2 in HCC.

**Supplementary Information:**

The online version contains supplementary material available at 10.1186/s13046-021-01838-5.

## Background

Hepatocellular carcinoma (HCC) is one of the most common malignancies worldwide [1], and is a highly lethal cancer because it is frequently diagnosed at the late stage [2]. More and more evidences indicate that the pathogenesis of HCC involves numbers of key molecules, including their protein expression and genetic and epigenetic changes [3]. However, the molecular mechanisms of HCC are not yet fully elucidated and still need to be further studied.

Long noncoding RNAs (lncRNAs) are defined as RNA longer than 200 nucleotides transcribed by RNA polymerase II and usually originated from intergenic regions [4]. LncRNAs can be capped, spliced, and polyadenylated and always show limited protein coding potential [5–7]. LncRNAs are emerging as a basic aspect of biology because of their ability to reprogram gene expression and influence different cellular functions including cell fate determination, cell cycle progression, apoptosis, and aging [8]. Their expression is tissue restricted, developmentally regulated, and can change under specific pathological conditions. Many lncRNAs have effects on a number of cancer progressions such as uncontrolled proliferation, evasion of cell death, metastasis formation and it has been researched that lncRNAs can function as oncogenes or tumor suppressors by influencing different signaling pathways [9]. Abnormal changes of lncRNAs have been indicated to have specific effects on the development of cancers, such as breast cancers [10], lung cancers [11], gastric cancers [12], and liver cancers [13]. The concrete mechanisms of lncRNAs in tumor progression need to be further researched.

LncRNA-SNHG14 was researched obsessing distinct functions in different cancer types [14]. Recently, Pu et al. have reported the upregulation of SNHG14 in HCC, which promoted HCC progression via regulating miR-4673/SOCS1. Ji et al. have also demonstrated that lncRNA SNHG14 promoted the progression of cervical cancer by regulating miR-206/YWHAZ. Collectively, these researches have shown that SNHG14 is relevant to HCC development and progression. Nevertheless, the underlying mechanisms remain to be further elucidated.

Here, we further analyzed the mechanisms and biological functions of SNHG14 in HCC. Moreover, we firstly reported the stimulative roles of SSR2 in HCC tumorigenesis and elucidated that SNHG14 accelerated the progression of HCC through the miR-876-5p/SSR2 axis.

## Material and methods

### Patients and tissue specimens

Human tumor and adjacent non-tumor tissues were collected from HCC patients underwent hepatectomy between December 2013 and December 2015 at the Hepatic Surgery Center, Tongji Hospital of Huazhong University of Science and Technology (HUST) (Wuhan, China). All procedures were approved by the Ethics Committee of Tongji Hospital, HUST and conducted according to the Declaration of Helsinki Principles. Prior written and informed consent was obtained from each patient.

## Cell lines and cell culture

HCC cell lines 97H was purchased from the Liver Cancer Institute of Fudan University. The 7702, HepG2 and Hep3B were purchased from China Center for Type Culture Collection (CCTCC, Wuhan, China). HLF was deposited in the Hepatic Surgery Center, Tongji Hospital. These cell lines were cultured in Dulbecco’s modified Eagle’s medium (Invitrogen) supplemented with 10% fetal bovine serum (Gibco, Grand Island, NY) and incubated in 5% CO2 at 37 °C.

### Cell transfection

The pcDNA3.1 vector (Invitrogen, USA) containing the full-length cDNA sequences of SNHG14 and short hairpin RNAs (shRNAs) targeting SNHG14 were provided by Tsingke Biological Technology (Beijing, China). The empty pcDNA3.1 vector and scramble shRNA or siRNA were utilized as negative controls. MiR-876-5p mimics and miR-876-5p inhibitors were designed by RiboBio (Guangzhou, China). All of the above reagents were transfected into cells via Lipo 2000 Transfection Reagent (Invitrogen) according to the manufacturer’s recommendations.

### Cell proliferation assay

Before analysis of cell proliferation, HLF and 97H cells were seeded into 96-well plates at a concentration of 1000 cells/well. Then proliferation was determined through the Cell Counting Kit-8 (CCK-8, Dojindo, Tokyo, Japan) according to the manufacturer’s protocol. All experiments were performed three times and the average percentages of cells were shown.

### Transwell cell migration and invasion assays

Cell migration assays were performed using a 24-well Transwell plate (pore size, 8 μm; Corning, NY, USA), according to the manufacturer’s protocol. For the Matrigel invasion assay, filters were precoated with 40 μl 1:4 mixture of Matrigel (BD Biosciences, NJ, USA) and DMEM without serum for 4 h at room temperature. Briefly, for invasion and migration assays, culture medium containing 10% FBS was added to the lower chambers and aliquots of 5 × 10^4^ cells in 100 μl serum-free medium were seeded into the upper chambers. After a 24 h incubation at 37 °C, non-migrated or non-invaded cells were removed by scraping the upper surface of the membranes with a cotton swab. Cells on the lower surface of the membranes were fixed with 4% paraformaldehyde at room temperature for 15 min and stained with 0.1% crystal violet at room temperature for 20 min. Cell numbers were counted under an optical microscope. Each experiment was repeated at least three times.

### RT–qPCR

FastPure Cell/Tissue Total RNA Isolation Kit (Vazyme Biotech Co., Ltd) was used to extract the total RNA from tissues and cells according to a modified version of the manufacturer’s protocol. The reverse transcription of lincRNA and mRNA was completed using a reverse-transcription system kit (Takara, Otsu, Japan), qPCR analysis was performed with a standard SYBR Green PCR kit (Toyobo Life Science, Osaka, Japan) according to the manufacturers’ protocols, GAPDH was used as the endogenous control for the detection of mRNA expression levels. And the reverse transcription of miRNA was using a miRNA First-Strand cDNA Kit (TIANGEN, Beijing, China), qPCR analysis was performed with miRcute Plus miRNA qPCR Kit (TIANGEN, Beijing, China), and U6 was used as the endogenous control for miRNA expression analysis. Relative quantification analysis was performed using the comparative CT (2^-ΔΔCT^) method. Each assay was repeated three times, independently of each other. Gene-specific primers used in this study were as follows: GAPDH-F: 5′-GACAAGCTTCCCGTTCTCAG-3′ and GAPDH-R: 5′-GAGTCAACGGATTTGGTCGT-3′; SNHG14-F: 5′-GGGTGTTTACGTAGACCAGAACC-3′ and SNHG14-R: 5′-CTTCCAAAAGCCTTCTGCCTTAG-3′; SSR2-F: 5′-TTGGCCACTTTCTCCTGGAT-3′ and SSR2-R: 5′-GAGAATTCACGTTGCCAGCA-3′.

### Dual-luciferase report assays

The entire 3′-untranslated region (UTR) of the SSR2 and the representative length of SNHG14 gene were cloned into the psiCHECKTM-2-vector (Promega, Madison, WI, USA) at a site immediately downstream of the Renilla luciferase gene. The mutations in the SSR2 3′-UTR and SNHG14 binding sites were generated with the Quick Change Site-Directed Mutagenesis kit (Vazyme Biotech Co., Ltd). About 1 × 10^5^ cells/well were seeded into 24-well plates for 24 h before transfection. Cells were co-transfected with 50 ng of the psiCHECKTM-2-vector and 50 nM of the miR-876-5p or mimic-NC using ExFect®2000 Transfection Reagent (Vazyme Biotech Co., Ltd). Cell lysates were prepared using Passive Lysis Buffer (Promega) 48 h after transfection, and luciferase activity was measured using the Dual-Luciferase Reporter Assay (Promega). Experiments were repeated three times.

### RNA immunoprecipitation (RIP)

Magna RIP™ RNA-Binding Protein Immunoprecipitation Kit (Millipore, Germany) was used to enrich Ago2 binding RNA. Cells with indicated transfection were harvested and then lysed in the lysis buffer. Then, cell lysates underwent incubation with RIP buffer which contained magnetic beads. The beads were conjugated with Ago-2 antibody (Abcam, UK) or anti-IgG (Abcam) as negative control. Further, the samples were digested applying Dnase I and Proteinase K. The enriched RNA was subjected to qRT-PCR.

### Western blot

Cells were lysed with RIPA buffer containing a protease inhibitor cocktail (MedChemExpress, USA) on ice for 30 min. Cell lysates were quantified using a BCA Protein Assay Kit (Thermo Fisher Scientific) and equal amounts (20 μg/lane) of protein were analyzed by 10% SDS-PAGE (Boster Biological Technology, Wuhan, China) and transfer to PVDF membranes (Roche). The membranes were blocked with 5% non-fat milk at 37 °C for 1 h and were incubated with primary antibodies prepared with 5% BSA (Wuhan Promoter Biological Co., LTD.) at 4 °C overnight. Subsequently, the membranes were incubated with HRP-conjugated goat anti-rabbit or goat anti-mouse immunoglobulin G secondary antibodies (Jackson ImmunoResearch Laboratories) for at 37 °C for 1 h. Finally, the ECL detection system (Bio-Rad Laboratories) was used for visualization. Image Lab™ 4.0 software (Bio-Rad Laboratories) was used to semi-quantify blots. SSR2 antibody was purchased from proteintech (#10278–1-AP; Wuhan, Hubei, China).

### Animal studies

Four–five weeks old male BALB/c nude mice were purchased from HFK (Beijing, China) and maintained at SPF conditions. All animal experiments were approved by the Ethics Committee of Tongji Hospital, HUST. The whole procedure was in accordance with the “Guide for the Care and Use of Laboratory Animals” (NIH publication 86–23 revised 1985). For Subcutaneous tumor model, 1 × 10^6^ logarithmically growing HLF cells stably expressing miR-NC and miR-876-5p were subcutaneously injected into nude mice. After 5 weeks, the nude mice were sacrificed, and the tumor tissues were stripped and weighed. Tumor volume was calculated using the formula: V (mm^3^) = 0.5 x L (mm) x W^2^ (mm^2^). For lung metastasis model, HCC cells (1 × 10^6^) were injected into the caudal veins of 5-week-old male BALB/C nude mice. Each group had five mice. All the mice groups were sacrificed 6 weeks after injection. The lungs of each mouse were separated and fixed for H&E staining. The average number of metastatic foci in each group was counted under a microscope.

### Statistical analysis

All statistical analyses were performed using SPSS 21.0 statistical software. Data are represented as the mean ± SEM. Quantitative data were performed by two-tailed Student t test, analysis of variance (ANOVA) with Bonferroni post hoc test, or Pearson’s correlation test when applicable. Categorical data were analyzed by χ2 test. A value of *P* < 0.05 was considered statistically significant.

## Results

### The high expression of SNHG14 in HCC tissues predicts poor prognosis

**Fig. 1:**
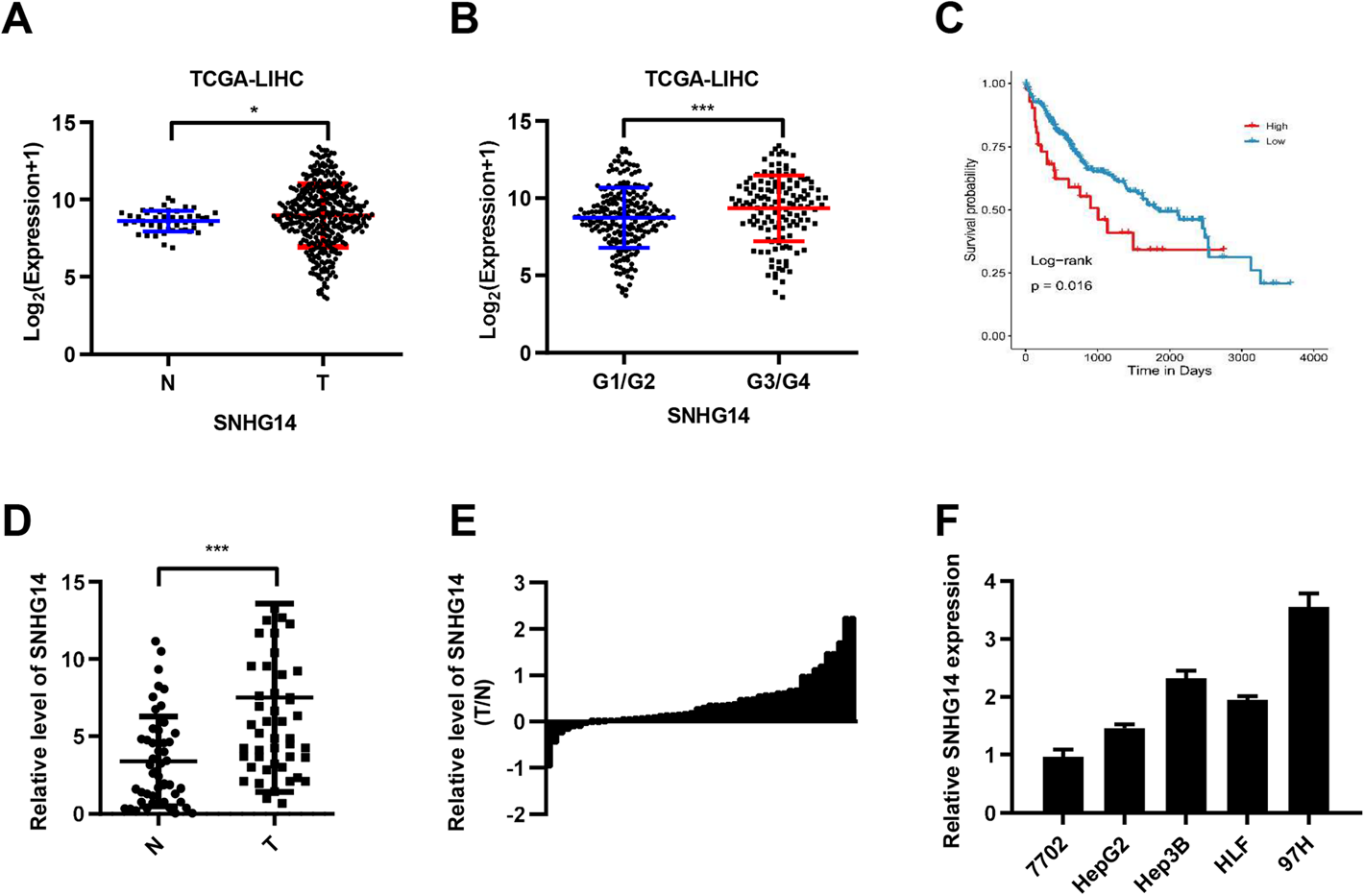
The high expression of SNHG14 in HCC tissues predicts poor prognosis. **a** Comparison the expression of SNHG14 in carcinoma and adjacent tissues in TCGA-LIHC. **b** Comparison the expression of SNHG14 in different grades (G1/G2 vs G3/G4) in TCGA-LIHC. **c** Prognostic difference of high and low expression of SNHG14. **d** Comparison the expression of SNHG14 in carcinoma and adjacent tissues. **e** SNHG14 expression in HCC and surrounding tissues were detected by rt-qPCR. And then the SNHG14 expression in HCC was normalized to their own adjacent nontumorous liver tissues. **f** RT-qPCR result of SNHG14 expression in different HCC cell lines. *P < 0.05, ***P < 0.001

SNHG14 is highly expressed in a variety of tumors (PMID: 31570691, PMID: 31273190, PMID: 30063126). To investigate its expression in HCC, we compared the expression of SNHG14 in HCC and adjacent tissues in TCGA. Overall, SNHG14 expression was higher in HCC tissues (Fig. 1a, *P* < 0.05). Further, we analyzed the expression of SNHG14 in HCC tissues with different grades, and the expression of SNHG14 was higher in carcinoma tissues with high grade (G3/G4) (Fig. 1b, *P* < 0.001). In order to verify the expression of SNHG14 in HCC and adjacent tissues, we tested the expression of SNHG14 in 66 pairs of samples and Chi-squared analysis indicated that high expression of SNHG14 was significantly associated with advanced BCLC stage (*P* = 0.047) in HCC (Supplementary Table 2). Consistent with the results in TCGA, the expression of SNHG14 was higher in cancer tissues (Fig. 1d and e, *P* < 0.001). In addition, high SNHG14 expression predicted poorer overall survival in TCGA (Fig. 1c, *P* < 0.05). Meanwhile, we selected several hepatocellular carcinoma cell lines for verification, which showed the highest expression in the most aggressive 97H cell line, and relatively low expression in the least aggressive 7702 cell line (Fig. 1f). These results suggest that SNHG14 plays a pro-oncogenic role in HCC.

### SNHG14 promotes proliferation, migration and invasion of HCC cells in vitro

**Fig. 2:**
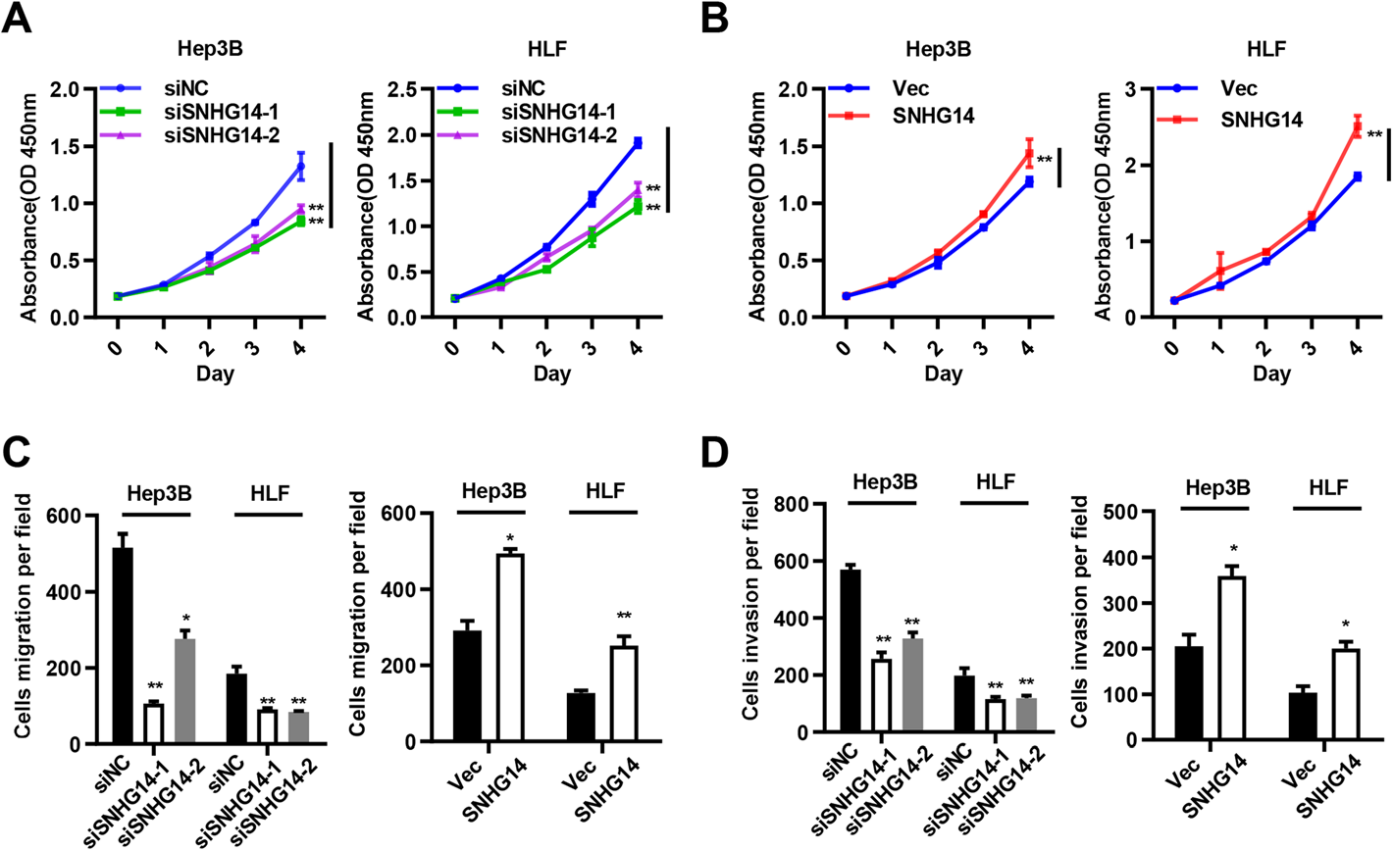
SNHG14 promotes proliferation, migration and invasion of HCC cells in vitro. **a-b** The viability of Hep3B and HLF cells transfected with siSNHG14 or pcDNA3.1/SNHG14 were detected by CCK8 assays. **c-d** Transwell migration and invasion assays were performed in SNHG14 silencing and overexpression cells. *P < 0.05, **P < 0.01

For determining the biological functions of SNHG14 in HCC, SNHG14 was silenced and overexpressed in Hep3B and HLF. qRT-PCR assay was used to verify the knockdown and overexpression efficiencies of si-SNHG14 and its overexpression in Hep3B and HLF. And compared with the vector group, the expression of SNHG14 was largely reduced in si-SNHG14 groups and increased in SNHG14 overexpression group (Fig. S1A and B). CCK8 assay indicated that SNHG14 knockdown inhibited the proliferation of Hep3B and HLF (Fig. 2a), whereas overexpression of SNHG14 greatly enhanced cell proliferation of Hep3B and HLF (Fig. 2b). Furthermore, transwell assay demonstrated that the migration and invasion abilities of Hep3B and HLF were obviously suppressed in cells transfected with si-SNHG14; when transfecting ectopic SNHG14, Hep3B and HLF gained increasing migration and invasion abilities (Figs. 2c, d and S1C, D). The above results illustrated that SNHG14 is able to promote the proliferation, migration and invasion of HCC cells.

### SNHG14 functions as a sponge for miR-876-5p in HCC cells

**Fig. 3:**
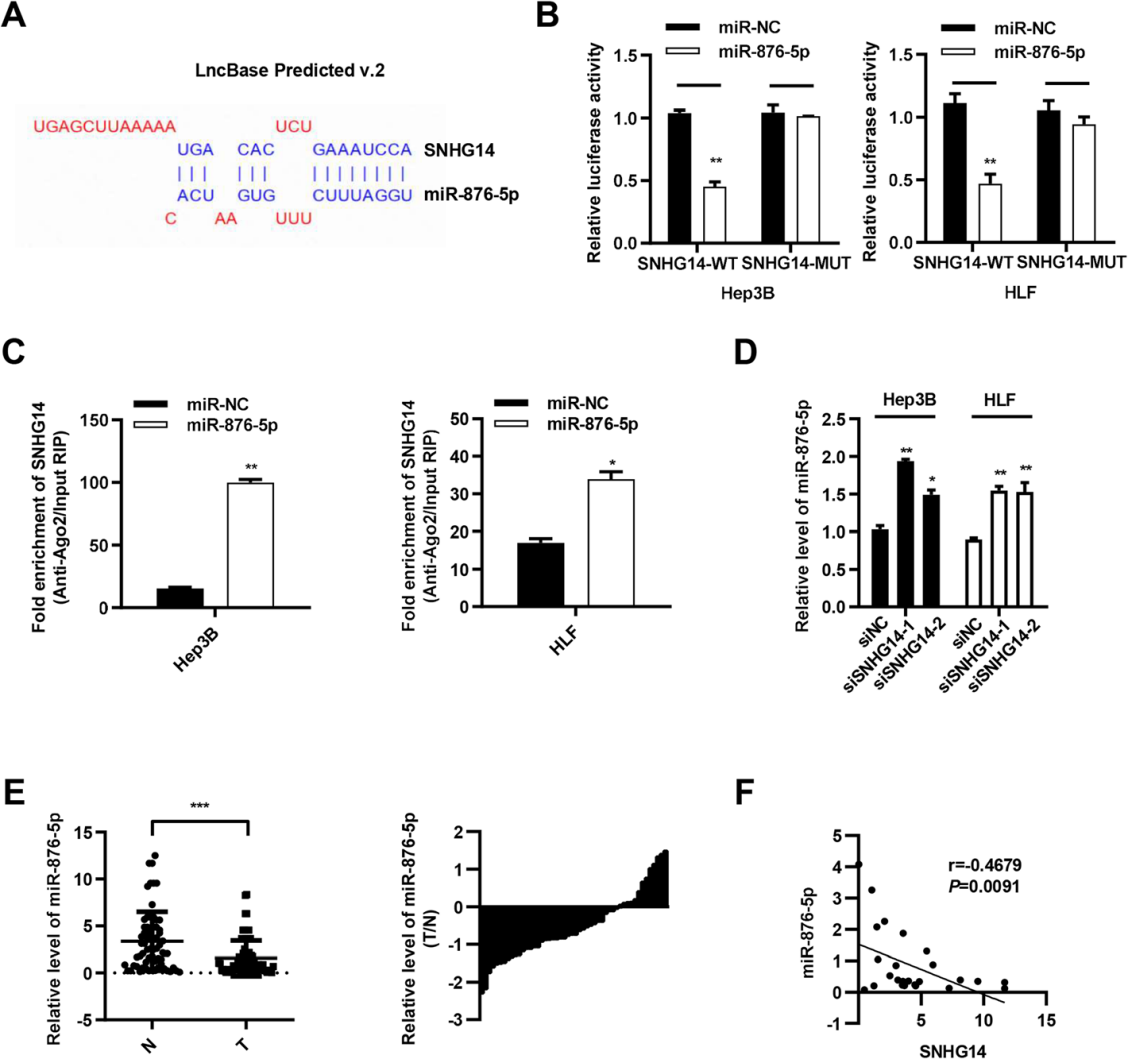
SNHG14 functions as a sponge for miR-876-5p in HCC cells. **a** The predicted binding sites of SNHG14 and miR-876-5p from LncBase Predicted v.2. **b** Relative luciferase activities of SNHG14-WT and SNHG14-MUT reporter were determined with miR-876-5p mimic and miR-NC co-transfection, respectively. **c** The enrichment of SNHG14 and miR-876-5p in RISC was identified with Ago2-RIP assay. **d** Quantitative PCR analysis of miR-876-5p after transfected with siSNHG14 and pcDNA3.1/SNHG14, respectively. **e** miR-876-5p in HCC specimens was analyzed by qPCR (*n* = 66). **f** Correlation between SNHG14 and miR-876-5p expression in paired HCC tissues. *P < 0.05, **P < 0.01, ***P < 0.001

We then studied the regulatory mechanism of SNHG14 in HCC. It has been reported that SNHG14, as a lncRNA, could act as a ceRNA by sponging miRNAs in some other diseases. Through bioinformatics prediction, LncBase Predicted v.2 (http://carolina.imis.athena-innovation.gr/diana_tools/web/index.php), we discovered that SNHG14 was likely to be a ceRNA for miR-876-5p (Fig. 3a) and their binding sites were indicated in Fig. 3a. MiR-876-5p has been reported to act as an anti-tumor role in many types of cancers. To confirm the interaction of miR-876-5p and SNHG14, dual luciferase reporter assay was performed and results indicated that the luciferase activity was obviously decreased when co-transfected with SNHG14-WT and miR-876-5p mimic. When co-transfected with mutant-type SNHG14 and miR-876-5p mimic, the activity displayed no difference compared with control group (Fig. 3b). RIP assay in Hep3B and HLF indicated that SNHG14 was more enriched in Ago2 pellet in miR-876-5p mimic group compared to that in miR-NC group (Fig. 3c). These results demonstrated that miR-876-5p could physically interacted with SNHG14 through the indicated binding sites. Furthermore, by qPCR analysis, we found that SNHG14 silencing resulted in the increased level of miR-876-5p in Hep3B and HLF cells (Fig. 3d). To further explore the correlation between miR-876-5p and SNHG14, we performed spearman’s correlation analysis and the result demonstrated that miR-876-5p expression was negatively correlated with SNHG14 expression in tumor tissues (r = − 0.4679, *P* = 0.0091, Fig. 3f). These findings showed that SNHG14 functions as a sponge of miR-876-5p in HCC cells.

### MiR-876-5p attenuates the proliferation, migration and invasion of HCC cells in vitro and in vivo

**Fig. 4:**
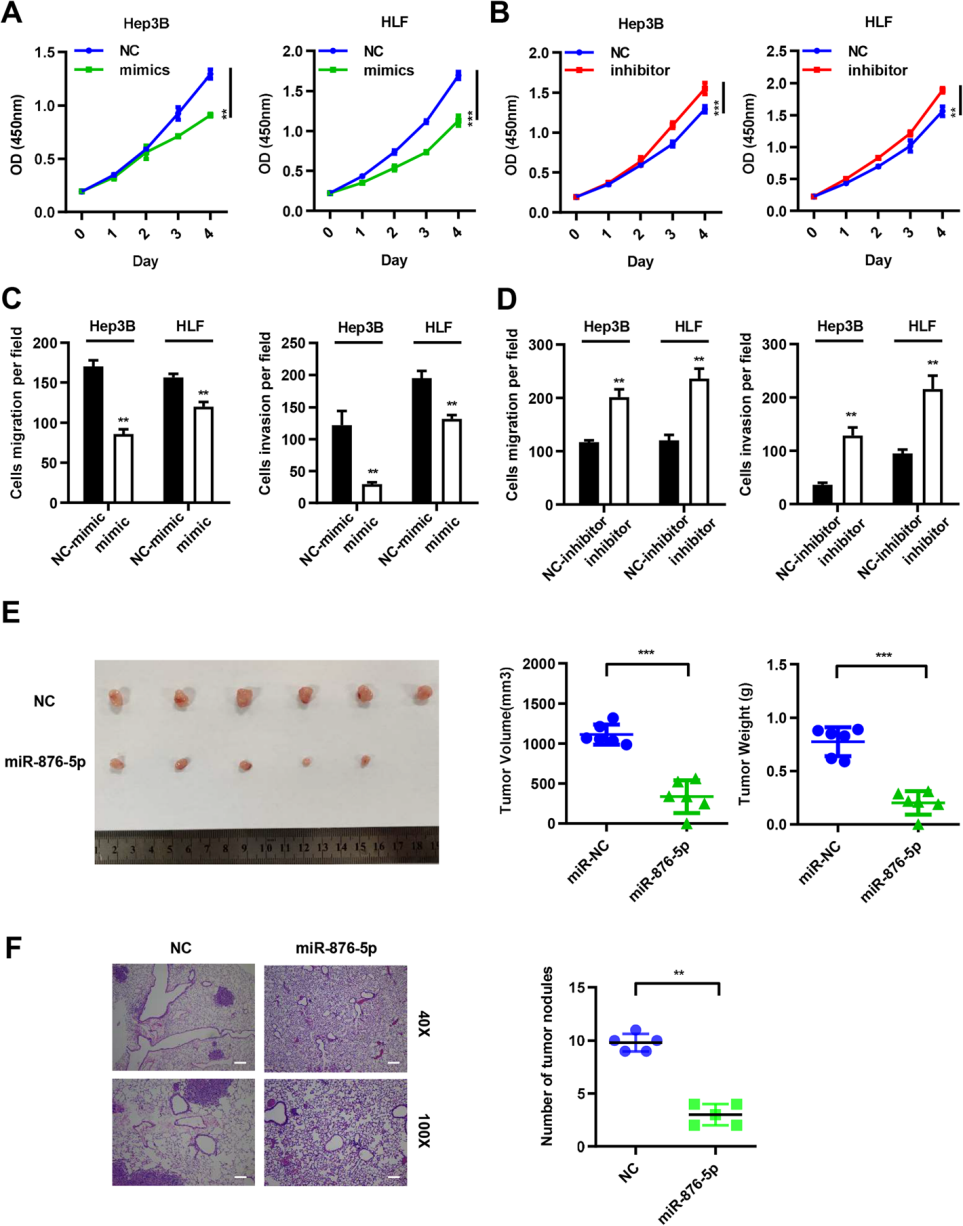
miR-876-5p attenuated the proliferation, migration and invasion of HCC cells in vitro and in vivo. **a-b** The CCK8 assays in HLF and Hep3B transfected with miR-876-5p mimic and miR-876-5p inhibitor, respectively. **c-d** Transwell assays were conducted to measure the migration and invasion abilities of HLF and Hep3B cells transfected with miR-876-5p mimic and miR-876-5p inhibitor respectively. **e** Images of tumors dissected from nude mice that were transplanted with miR-876-5p-overexpressed HLF cells and its negative control HLF cells. And tumor volume and tumor weight in these two groups were quantified. **f** The number of lung metastatic tumor nodules in these two groups were quantified. Scale bars: 40x = 250 um; 100x = 100 um. **P < 0.01, ***P < 0.001

In order to validate the roles of miR-876-5p in HCC, we examined the miR-876-5p expression in HCC patients (Supplementary Table 1). We found that miR-876-5p expression in tumor tissues was obviously downregulated in comparison to that in adjacent non-tumor tissues (Fig. 3e), and Chi-squared analysis indicated that lower expression of miR-876-5p was significantly associated with lower differentiation (*P* = 0.030) and advanced BCLC stage (*P* = 0.044) in HCC (Supplementary Table 3). Then CCK8 assay was performed and the result showed that miR-876-5p largely inhibited the proliferation ability in Hep3B and HLF (Fig. 4a and b). In addition, transwell assay indicated that miR-876-5p mimic significantly reduced the migration and invasion abilities (Figs. 4c and S2A), meanwhile, miR-876-5p inhibitor increased these abilities in Hep3B and HLF cells (Figs. 4d and S2B). Next, a xenograft tumor model was performed to demonstrate the role of miR-876-5p in vivo. We established miR-876-5p stably overexpression cell models and injected HLF-oemiR-876-5p cells and control cells subcutaneously in the flank of nude mice to evaluate the effect of miR-876-5p on HCC tumorigenicity. Consistent with our in vitro observations, overexpression of miR-876-5p resulted in significant decrease of tumor volume and tumor weight (Fig. 4e); for lung metastasis model, HLF cells stably overexpressing miR-876-5p were injected into the caudal veins of 5-week-old male BALB/C nude mice. All the mice groups were sacrificed 6 weeks after injection. The lungs of each mouse were separated and fixed for H&E staining and we found that the number of metastatic tumor nodules in miR-876-5p group in the lungs was less than that in control group (Fig. 4f). Collectively, these results suggested that miR-876-5p inhibited the proliferation, migration and invasion of HCC in vitro and in vivo.

### SSR2 is a downstream target of miR-876-5p

**Fig. 5:**
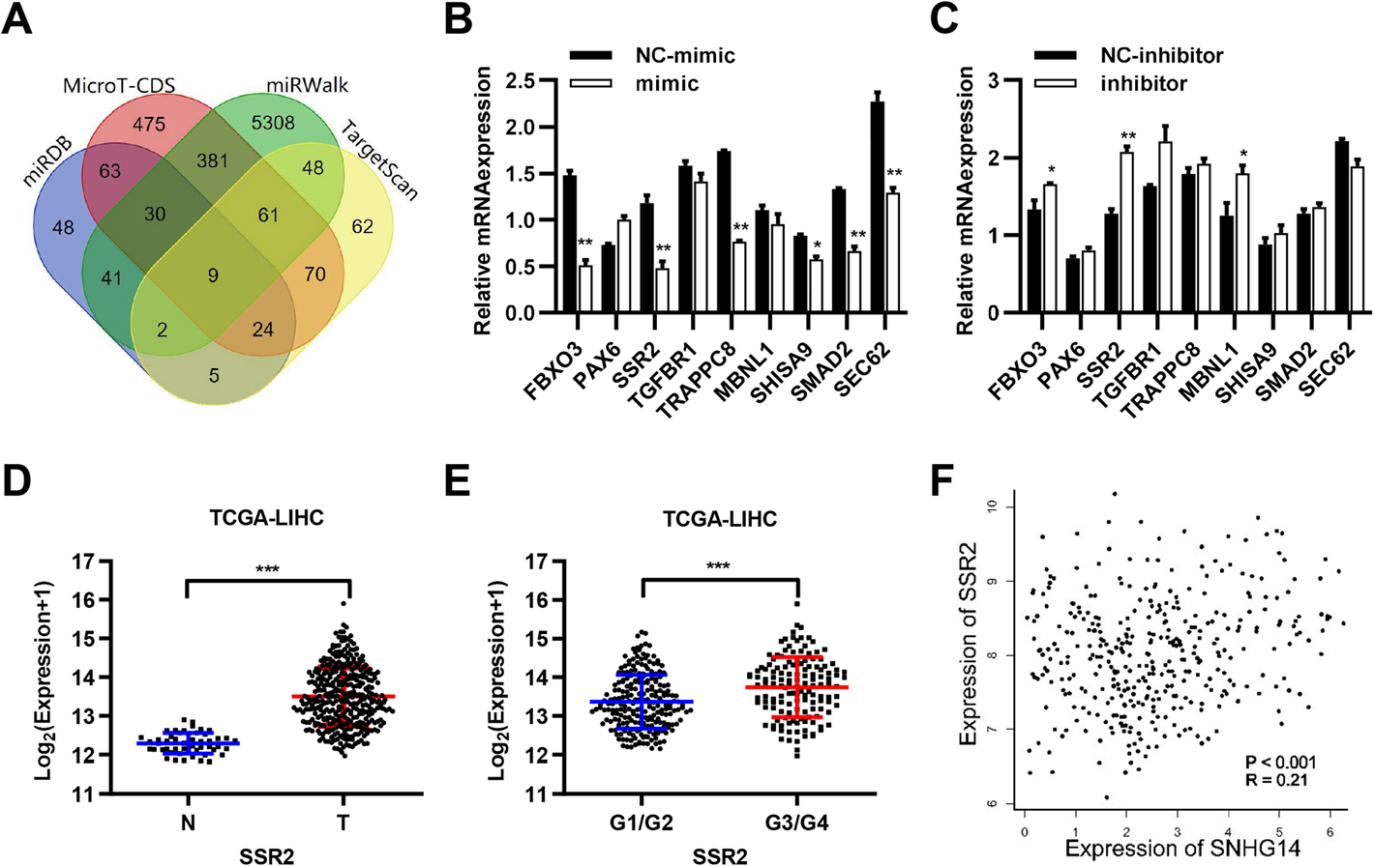
SSR2 is a downstream target of miR-876-5p. **a** The predicted results of miR-876-5p downstream target genes from four databases. **b-c** Relative expression of the nine downstream target genes when stimulated with miR-876-5p mimic or miR-876-5p inhibitor through RT-qPCR analysis. **d** Comparison the expression of SSR2 in carcinoma and adjacent tissues in TCGA-LIHC. **e** Comparison the expression of SSR2 in different grades (G1/G2 vs G3/G4) in TCGA-LIHC. **f** The correlation of the expression of SNHG14 and SSR2 in TCGA-LIHC. *P < 0.05, **P < 0.01, ***P < 0.001

To identify the regulatory mechanisms of miR-876-5p in HCC, the potential target genes of miR-876-5p were predicted by four bioinformatics tools (miRDB, MicroT, miRWalk, and TargetScan) and then we found nine candidate genes (Fig. 5a). To verify the research target gene, we used qPCR to detect the regulation of these genes by miR-876-5p mimic and its inhibitor (Fig. 5b and c), and we analyzed the correlation between candidate genes and SNHG14 (Fig. S3). Taken together, SSR2 was chosen as our downstream target gene. From the TCGA database, SSR2 expression was also higher in HCC tissues (Fig. 5d). Moreover, SSR2 expression was much higher in the patients in G3/G4 grades compared to patients in G1/G2 grades (Fig. 5e). The correlation analysis indicated that SSR2 positively correlated with lncRNA SNHG14 (Fig. 5f). Above all, these results showed that SSR2 is a downstream target of miR-876-5p in HCC.

### miR-876-5p inhibited SSR2 expression through binding to its 3’UTR

**Fig. 6:**
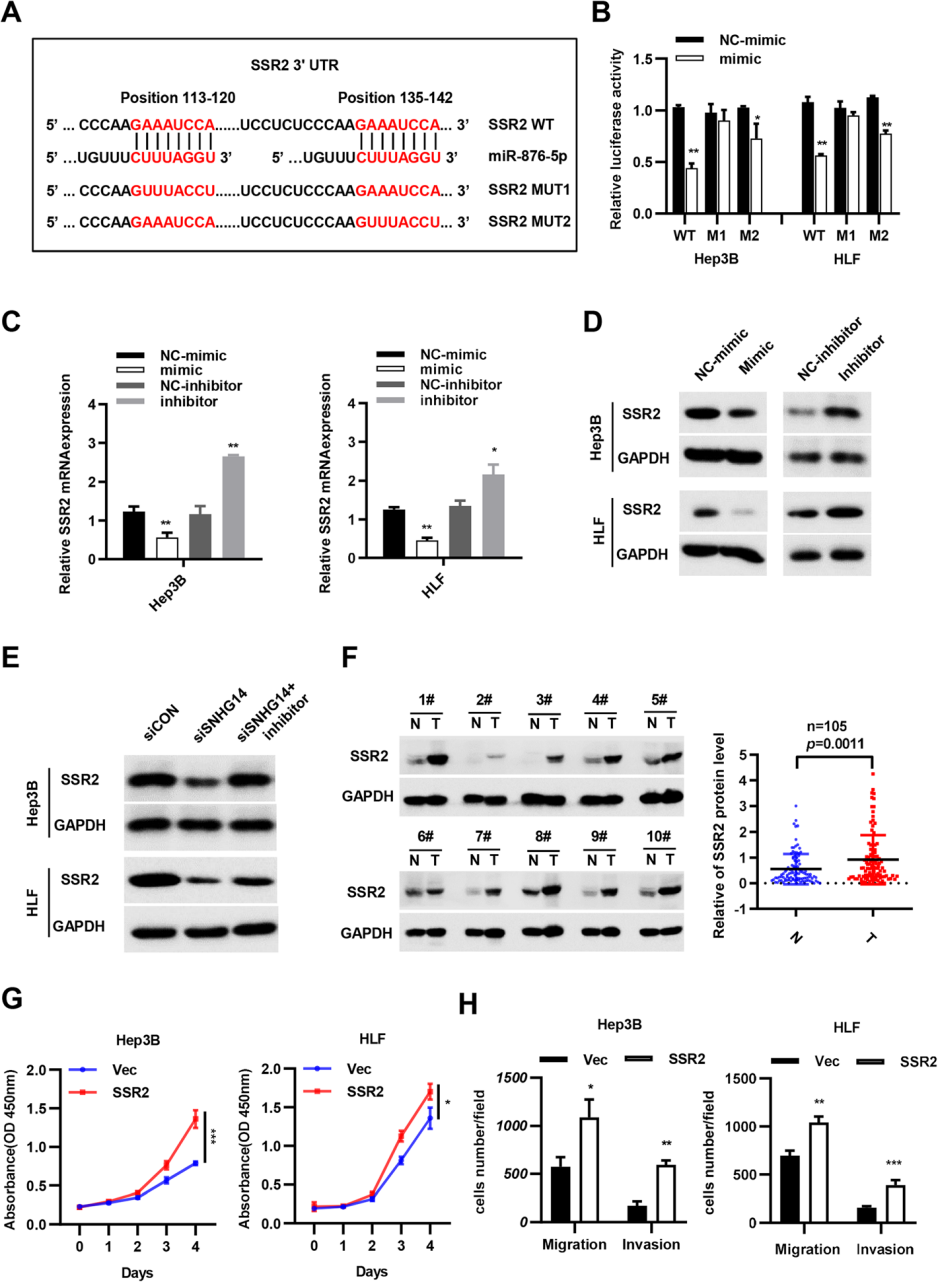
miR-876-5p inhibited SSR2 expression through binding to its 3’UTR. **a** Schematic view of miR-876-5p putative binding site in the WT and MUT 3′ UTR of SSR2. **b** Luciferase activity assays were conducted in Hep3B and HLF cells transfected with WT and MUT 3′ UTR of SSR2 luciferase report plasmids with miR-876-5p mimics. **c** Quantitative PCR analysis of SSR2 mRNA expression after transfected with miR-876-5p mimic or miR-876-5p inhibitor. **d** Western blot result of SSR2 protein levels after transfected with miR-876-5p mimic or miR-876-5p inhibitor. **e** SSR2 protein levels in Hep3B and HLF after SNHG14 knockdown with or without miR-876-5p inhibitor. **f** SSR2 protein levels were demonstrated in HCC clinical patient tissues (*n* = 105). **g** The viability of Hep3B and HLF cells transfected with pcDNA3.1/SSR2 were detected by CCK8 assays. **f** Transwell migration and invasion assays were performed in SSR2 overexpression cells. *P < 0.05, **P < 0.01

SSR2 was reported to be a member of the chromatin structure remodeling complex (RSC), which took part in transcription regulation and nucleosome positioning. And it required for the positive and negative regulation of gene expression of many genes. But its roles in HCC progression remained to be researched. It was already known that microRNA always targeted the 3’UTR of mRNAs, so we analyzed the sequences of miR-876-5p and 3’UTR of SSR2. As a result, we predicted two binding sites and constructed two mutation plasmids in psiCHEK2, respectively (Fig. 6a). Through dual luciferase reporter assay, we found that position 113–120 nt in the 3’UTR of SSR2 was the concrete sites combining with miR-876-5p (Fig. 6b). Moreover, qPCR and western blot analyses indicated that miR-876-5p mimic suppressed and miR-876-5p inhibitor promoted SSR2 expression, respectively (Fig. 6c and d). Collectively, these results demonstrated that SSR2 was the downstream target of miR-876-5p. To verify the relationship among SNHG14, miR-876-5p and SSR2, we performed rescue experiments. Western blot assay exhibited that SSR2 expression was decreased in Hep3B and HLF transfected with si-SNHG14. However, the cotransfection of miR-876-5p inhibitor and si-SNHG14 rescued the repressive role of si-SNHG14 in the expression of SSR2 (Fig. 6e). Additionally, 105 pairs of HCC tissues indicated that SSR2 was upregulated in HCC tissues compared with adjacent non-tumor tissues (Figs. 6f and S4, S5). Then CCK8 assay was performed and the result showed that SSR2 largely enhance the proliferation ability in Hep3B and HLF (Fig. 6g). In addition, transwell assay indicated that SSR2 significantly enhanced the migration and invasion abilities of HCC cells (Figs. 6h and S6).

### SNHG14 promotes HCC progression by regulating miR-876-5p/SSR2 axis

**Fig. 7:**
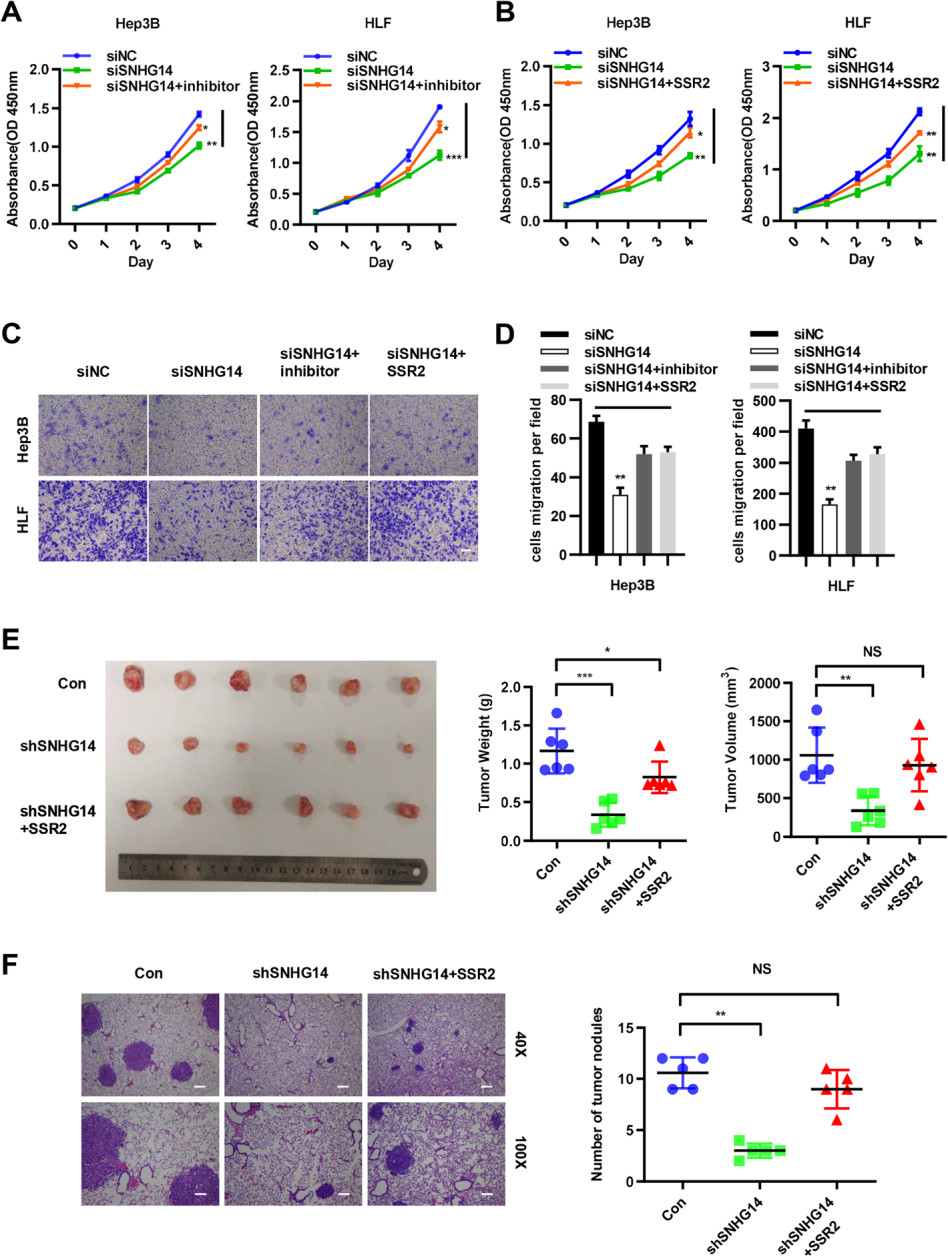
SNHG14 promotes HCC progression by regulating miR-876-5p/SSR2 axis. **a-b** The CCK8 assays in HLF and Hep3B with co-transfection of siSNHG14 and miR-876-5p inhibitor or pcDNA3.1/SSR2. **c-d** The transwell assays in HLF and Hep3B with co-transfection of siSNHG14 and miR-876-5p inhibitor or pcDNA3.1/SSR2. Scale bars: 100x = 100 um. **e** Images of tumors dissected from nude mice that were transplanted with SNHG14 knockdown HLF cells, SNHG14 knockdown plus SSR2 overexpression HLF cells and its negative control HLF cells. And tumor volume and tumor weight in these two groups were quantified. **f** The number of lung metastatic nodules in these three groups were quantified. Scale bars: 40x = 250 um; 100x = 100 um. *P < 0.05, **P < 0.01, ***P < 0.001

In order to further explore if SNHG14 promoted HCC progression by targeting miR-876-5p/SSR2 axis, we first checked whether SNHG14 regulated the expression of SSR2 independent of miR-876-5p through RIP analysis. The results showed that SNHG14 regulates SSR2 to promote HCC progression dependent on miR-876-5p (Fig. S7). Next, we co-transfected miR-876-5p inhibitor or pcDNA3.1/SSR2 with SNHG14 silencing in Hep3B and HLF cells. And CCK8 assays demonstrated that knockdown of SNHG14 inhibited the proliferative ability of Hep3B and HLF. Furthermore, miR-876-5p inhibitor partly restored the suppressive effects of SNHG14 silencing (Fig. 7a). Similarly, SSR2 overexpression additionally could also attenuate the repressive roles of SNHG14 silencing (Fig. 7b). From the transwell assays, including migration and invasion experiments, we obtained the similar results (Fig. 7c and d). Next, a xenograft tumor model was performed to demonstrate the role of SSR2 in the absence of SNHG14 in vivo. We established shSNHG14 stable cell lines and stably overexpressed SSR2 in the base of shSNHG14 cell lines. Then we injected HLF-shSNHG14, HLF-shSNHG14-SSR2 cells and control cells subcutaneously in the flank of nude mice to evaluate the effect of SSR2 on HCC tumorigenicity. Consistent with our in vitro observations, knocking down of SNHG14 resulted in significant decrease of tumor volume and tumor weight; while the overexpression of SSR2 could partially restore the inhibitor effects of shSNHG14 (Fig. 7e). As for lung metastasis model, these three kinds of cells were injected into the caudal veins of 5-week-old male BALB/C nude mice. Six weeks after injection, the lungs of each mouse were separated and fixed for H&E staining. From the H&E results, we found that the number of metastatic tumor nodules in shSNHG14 group was the least and SSR2 overexpression could partially restore the tumor number in lungs (Fig. 7f). In summary, we demonstrated that SNHG14 promotes HCC progression through regulating miR-876-5p/SSR2 axis.

## Discussion

Many researches have demonstrated that lncRNAs are of great importance in the tumorigenesis in many tumors [15, 16], such as bladder cancer [17], breast cancer [18], cervical cancer [19] and liver cancer [20, 21]. In human cancers, the regulatory mechanisms of some famous lncRNAs have been defined. However, mountains of lncRNAs have not been widely studied. Long non-coding RNA SNHG14, in recent studies, has been proved to promote proliferation and metastasis in many cancers. For example, SNHG14 promoted cell migration, invasion, proliferation and resistance to cell apoptosis in gastric cancer via miR-145/SOX9 axis. Pu et al. reported the SNHG14 dysregulation in HCC through bioinformatics analysis and found that SNHG14 promoted cell proliferation and inhibited cell apoptosis in HCC cells partially through miR-4673/SOCS1 axis. In this study, we further confirmed that SNHG14 was upregulated in our collected HCC specimens. Additionally, our data suggested that SNHG14 expression was associated with HCC stage and survival probability. Through CCK8 and transwell analysis, we found that knockdown of SNHG14 inhibited the proliferation and metastasis in HCC cell lines. In vitro experiments and clinical evidences demonstrated that SNHG14 promoted HCC progression.

MicroRNAs (miRNAs) are short non-coding RNA molecules with 20–24 nucleotides, and could regulate many tumorous progresses [22–26]. And lncRNAs could function as ceRNAs by sponging miRNAs to play its roles. What’s more, miRNAs always bound to 3’UTR of target mRNAs to degradeits downstream target genes [22, 27–33]. MiR-876-5p was reported to have different roles in different types of tumorigenesis. For example, PITPNA-AS1 abrogated the inhibition of miR-876-5p on WNT5A to facilitate HCC progression [34, 35]. MicroRNA-876-5p inhibited cell proliferation, migration and invasion by targeting c-Met in osteosarcoma [36]. In our study, we identified a new downstream target of miR-876-5p, SSR2. Moreover, through luciferase reporter assay, RIP, RNA pull down and qRT-PCR, we confirmed that SNHG14 could physically bind to miR-876-5p and its expression was negatively regulated by SNHG14. Combined with our clinical cohort, we ascertained the repressive role of miR-876-5p in HCC patients.

SSR2 was reported to be a component of the chromatin structure remodeling complex (RSC), which is involved in transcription regulation and nucleosome positioning. It controls particularly membrane and organelle development genes. As a part of the SWI/SNF complex, SSR2 functions as an ATP-dependent chromatin remodeling complex, required for the positive and negative regulation of gene expression of a large number of genes. It changes chromatin structure by altering DNA-histone contacts within a nucleosome, resulting in a change in nucleosome position, thus facilitating or repressing binding of gene-specific transcription factors [37, 38]. In this study, we explored its possible roles in HCC progression and its relationship with SNHG14. From our clinical specimens, the expression of SSR2 was much higher in tumor tissues compared with that in adjacent non-tumor tissues. Through restoration experiments, we found that SSR2 knockdown could partially attenuate the biological function of SNHG14. Collectively, we could conclude that SNHG14 exerted its tumor-promoting effects through miR876-5p/SSR2 axis in a ceRNA model. However, there still remained problems to be solved. The biological functions of SSR2 and its underlying mechanisms in HCC progression remain to be further elucidated.

## Conclusions

In conclusion, our study demonstrated that SNHG14 promotes the development of HCC by sponging miR-876-5p and increasing the expression of SSR2. It highlights the significant role of the SNHG14/miR-876-5p/SSR2 axis in HCC progression, suggesting that SNHG14 may serve as a potential biomarker and therapeutic target for HCC.

## Supplementary Information

**Additional file 1: Supplementary Figure 1.** SNHG14 promotes proliferation, migration and invasion of HCC cells in vitro. (A-B) The SNHG14 relative expression level was examined after transfected with siSNHG14 or pcDNA3.1/SNHG14 through RT-qPCR analysis. (C-D) The migration and invasion images of HLF and Hep3B cells transfected with siSSNHG14 or pcDNA3.1/SNHG14. *P < 0.05, **P < 0.01.

**Additional file 2: Supplementary Figure 2.** miR-876-5p attenuated the proliferation, migration and invasion of HCC cells in vitro and in vivo. (A-B) The migration and invasion images of HLF and Hep3B cells transfected miR-876-5p mimic or miR-876-5p inhibitor.

**Additional file 3: Supplementary Figure 3.** The correlation between the expression of the indicated genes and SNHG14 in HCC patients.

**Additional file 4: Supplementary Figures 4-5.** The expression of SSR2 are elevated in HCC tissues compared with adjacent tissues.

**Additional file 5: Supplementary Figure 6.** SSR2 promotes migration and invasion of HCC cells in vitro.

**Additional file 6: Supplementary Figure 7.** SNHG14 regulates SSR2 to promote HCC progression dependent on miR-876-5p. (A) SSR2 protein levels in HLF after transfecting with miR-876-5p mimic and with or without SNHG14. (B) RIP assay was performed to identify the combination between SNHG14 and SSR2.

**Additional file 7: Supplementary Table 1.** Clinicopathologic characteristics of patients with hepatocellular carcinoma.

**Additional file 8: Supplementary Table 2.** Correlation between relative SNHG14 expression and clinicopathologic characeristics in HCC patients (*n* = 66).

**Additional file 9: Supplementary Table 3.** Correlation between relative has-miR-876-5p expression and clinicopathologic characeristics in HCC patients (*n* = 66).

## Data Availability

All the data and materials supporting the conclusions were included in the main paper.

## Abbreviations

HCC: Hepatocellular carcinoma
LncRNA: Long noncoding RNA
TCGA: The Cancer Genome Atlas
qRT-PCR: Quantitative real-time PCR
ceRNA: Competing endogenous RNA
3’UTR: 3’Untranslated region
RIP: RNA immunoprecipitation
CCK8: Cell counting kit 8
PVDF: Polyvinylidene fluoride
