# Supplementary figures and images for: Long noncoding RNA SNHG14 promotes hepatocellular carcinoma progression by regulating miR-876-5p/SSR2 axis
Source: J Exp Clin Cancer Res. 2021 Jan 23;40:36. doi: 10.1186/s13046-021-01838-5 (PMC7824933; doi:10.1186/s13046-021-01838-5)

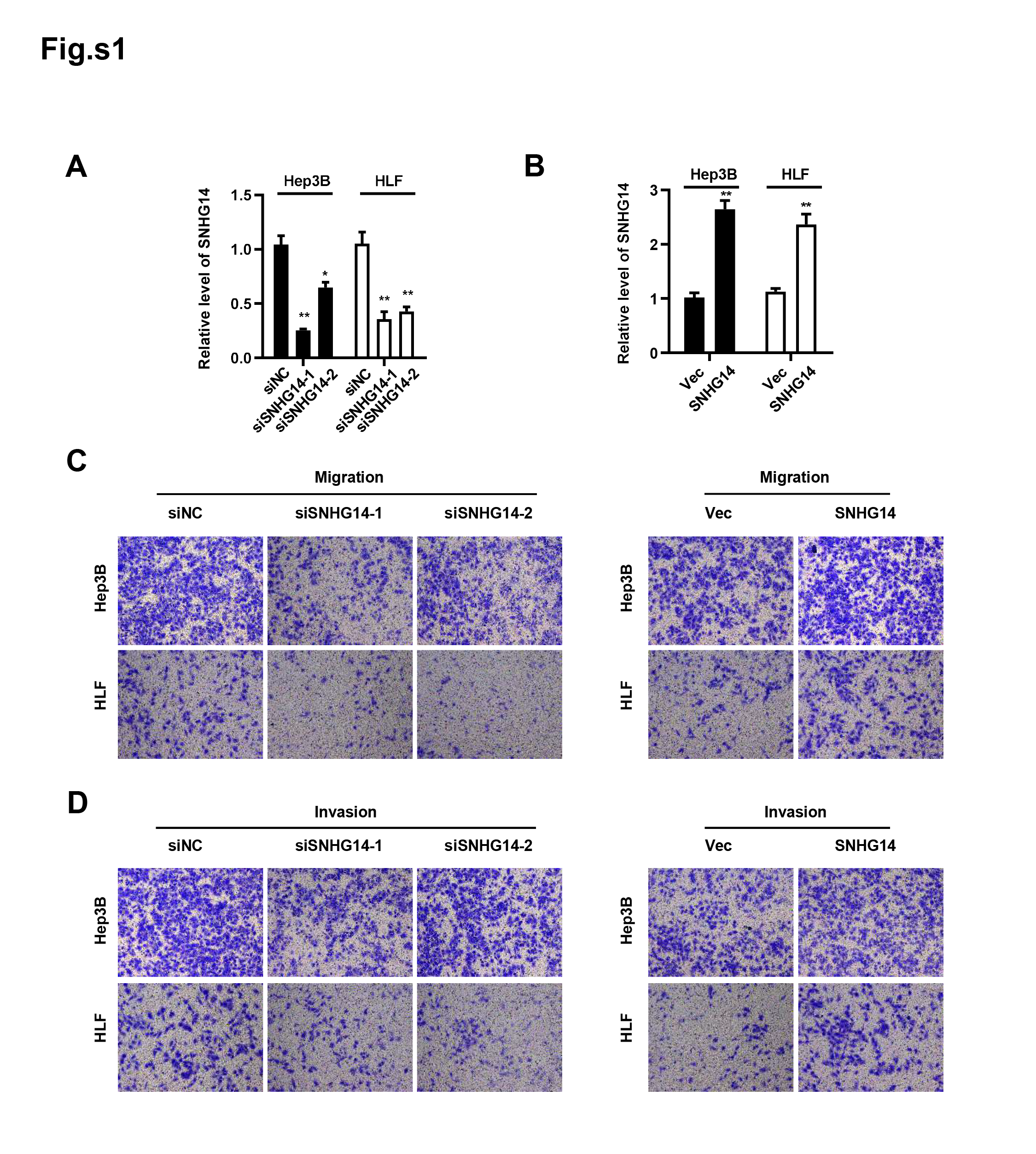

Supplement: Supplementary file 1 — Additional file 1: Supplementary Figure 1. SNHG14 promotes proliferation, migration and invasion of HCC cells in vitro. (A-B) The SNHG14 relative expression level was examined after transfected with siSNHG14 or pcDNA3.1/SNHG14 through RT-qPCR analysis. (C-D) The migration and invasion images of HLF and Hep3B cells transfected with siSSNHG14 or pcDNA3.1/SNHG14. *P < 0.05, **P < 0.01. [file 13046_2021_1838_MOESM1_ESM.tif]

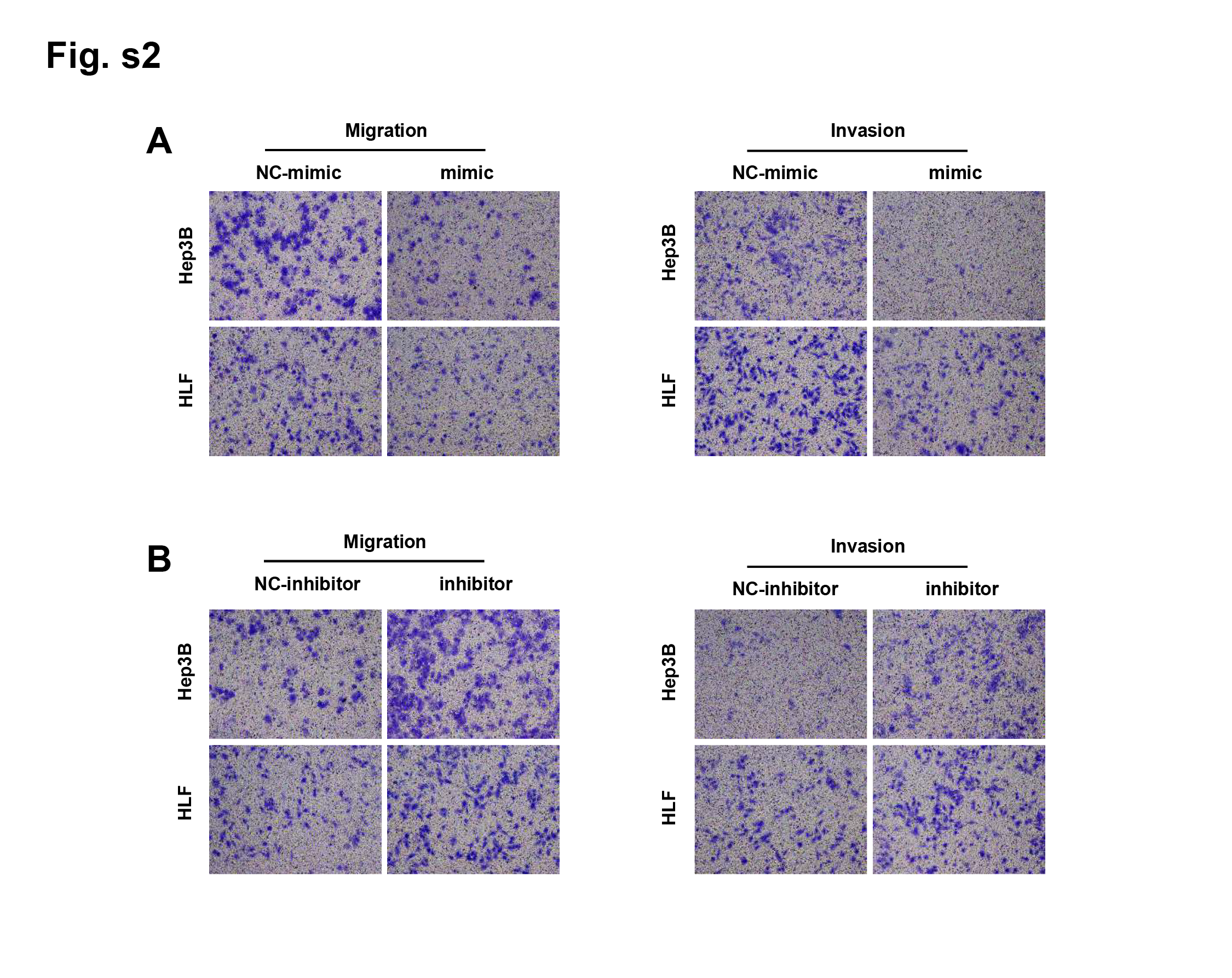

Supplement: Supplementary file 2 — Additional file 2: Supplementary Figure 2. miR-876-5p attenuated the proliferation, migration and invasion of HCC cells in vitro and in vivo. (A-B) The migration and invasion images of HLF and Hep3B cells transfected miR-876-5p mimic or miR-876-5p inhibitor. [file 13046_2021_1838_MOESM2_ESM.tif]

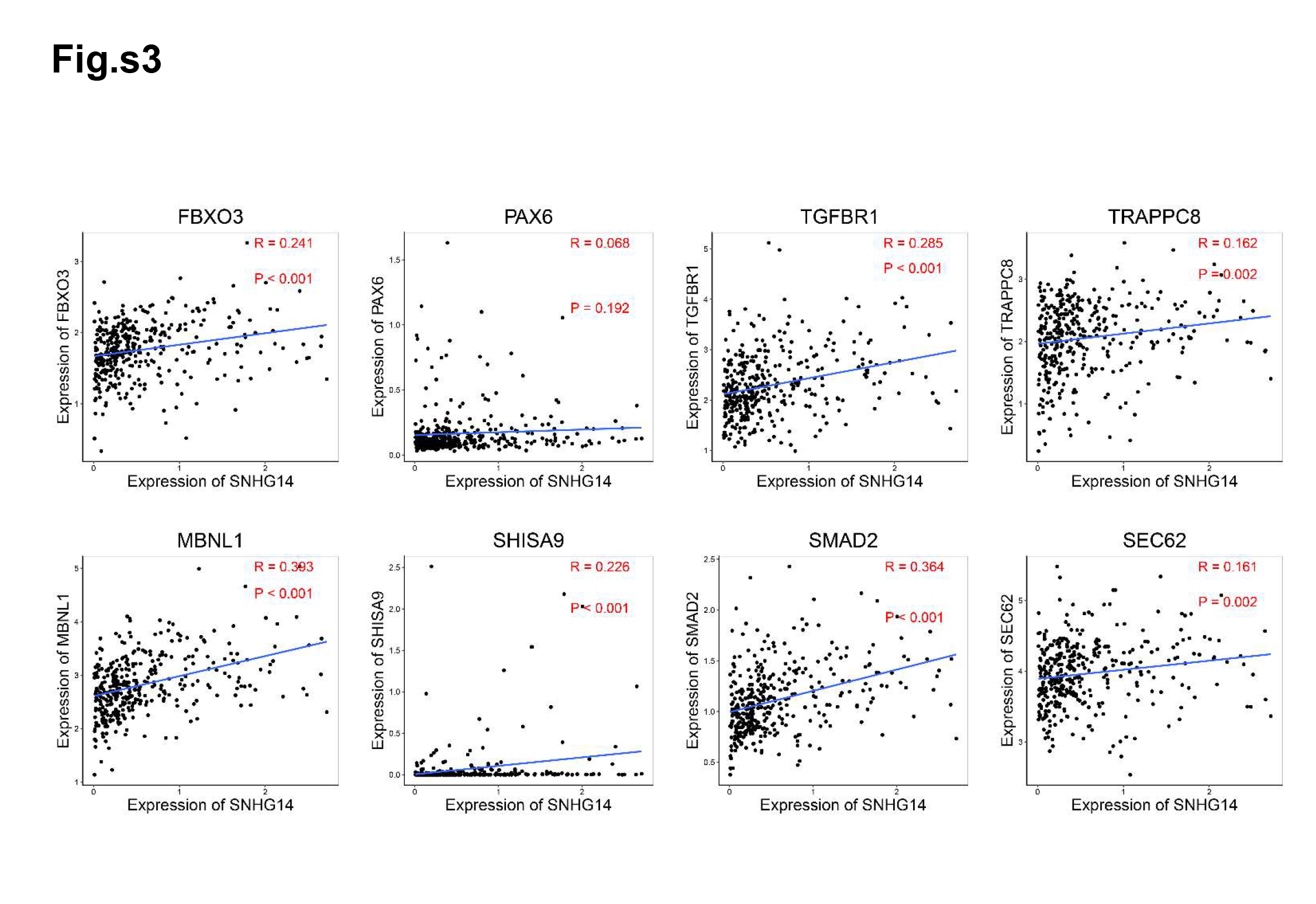

Supplement: Supplementary file 3 — Additional file 3: Supplementary Figure 3. The correlation between the expression of the indicated genes and SNHG14 in HCC patients. [file 13046_2021_1838_MOESM3_ESM.tif]

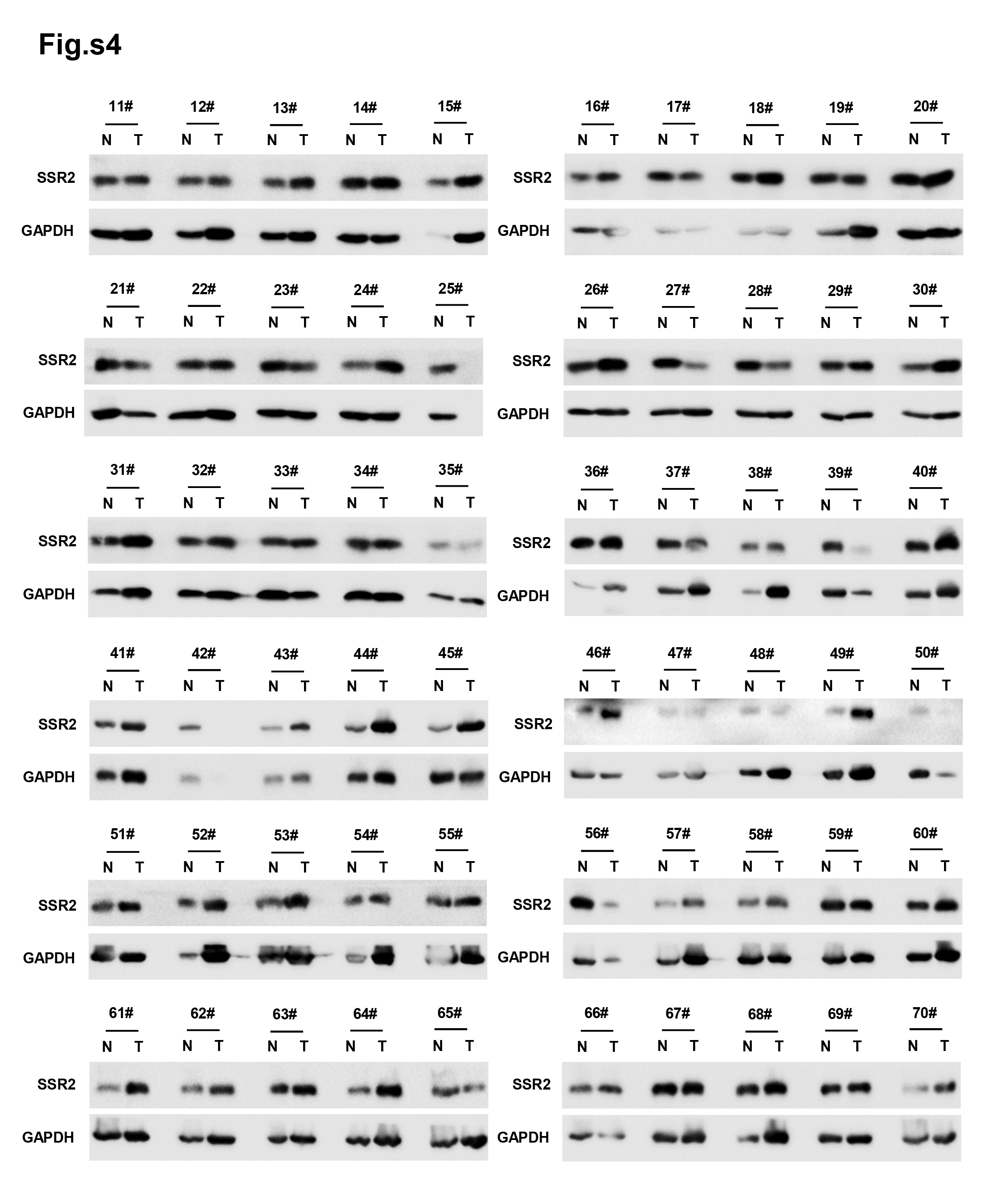

Supplement: Supplementary file 4 — Additional file 4: Supplementary Figures 4-5. The expression of SSR2 are elevated in HCC tissues compared with adjacent tissues. [file 13046_2021_1838_MOESM4_ESM.zip › Fig.s4.tif]

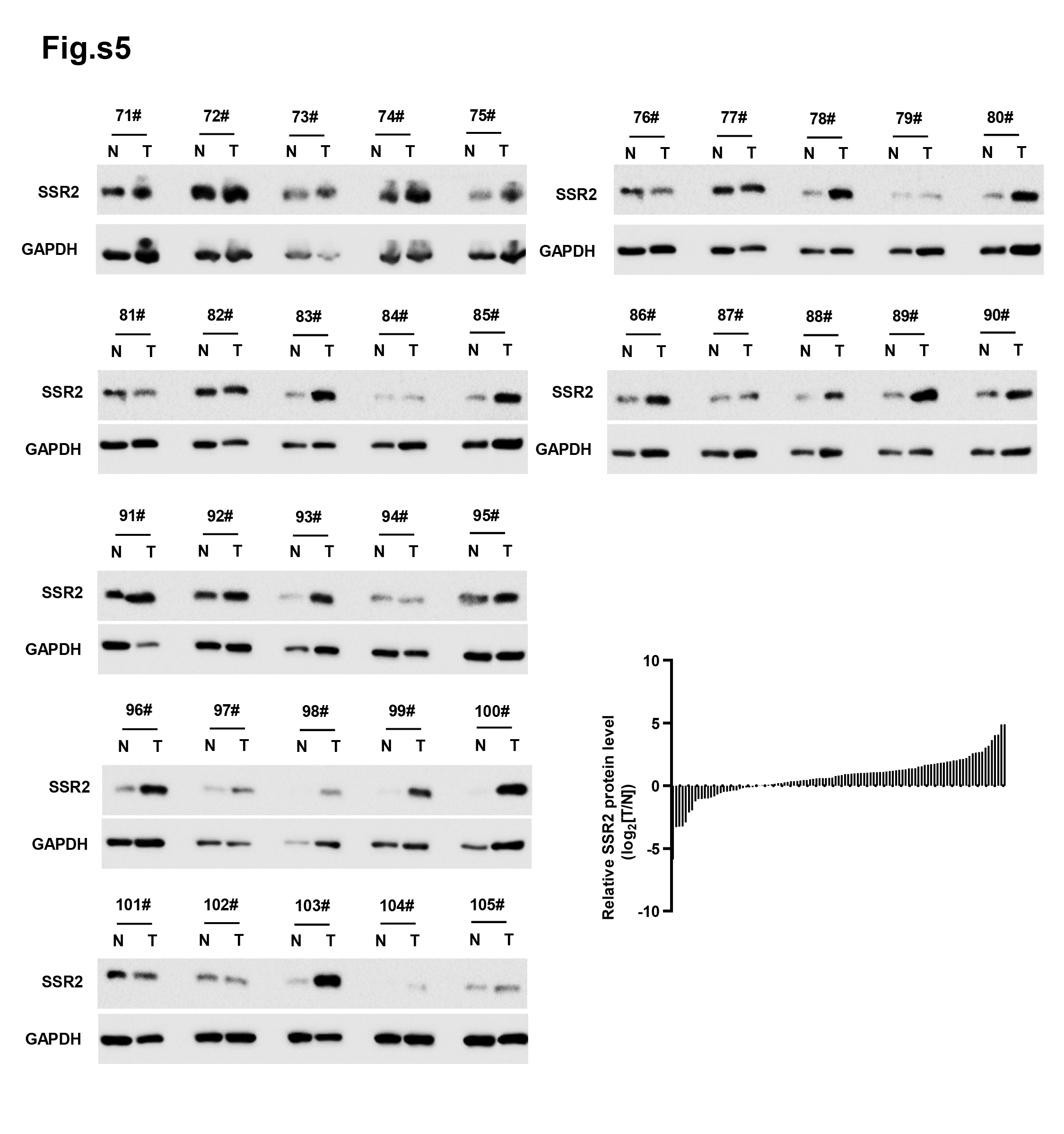

Supplement: Supplementary file 4 — Additional file 4: Supplementary Figures 4-5. The expression of SSR2 are elevated in HCC tissues compared with adjacent tissues. [file 13046_2021_1838_MOESM4_ESM.zip › Fig.s5.tif]

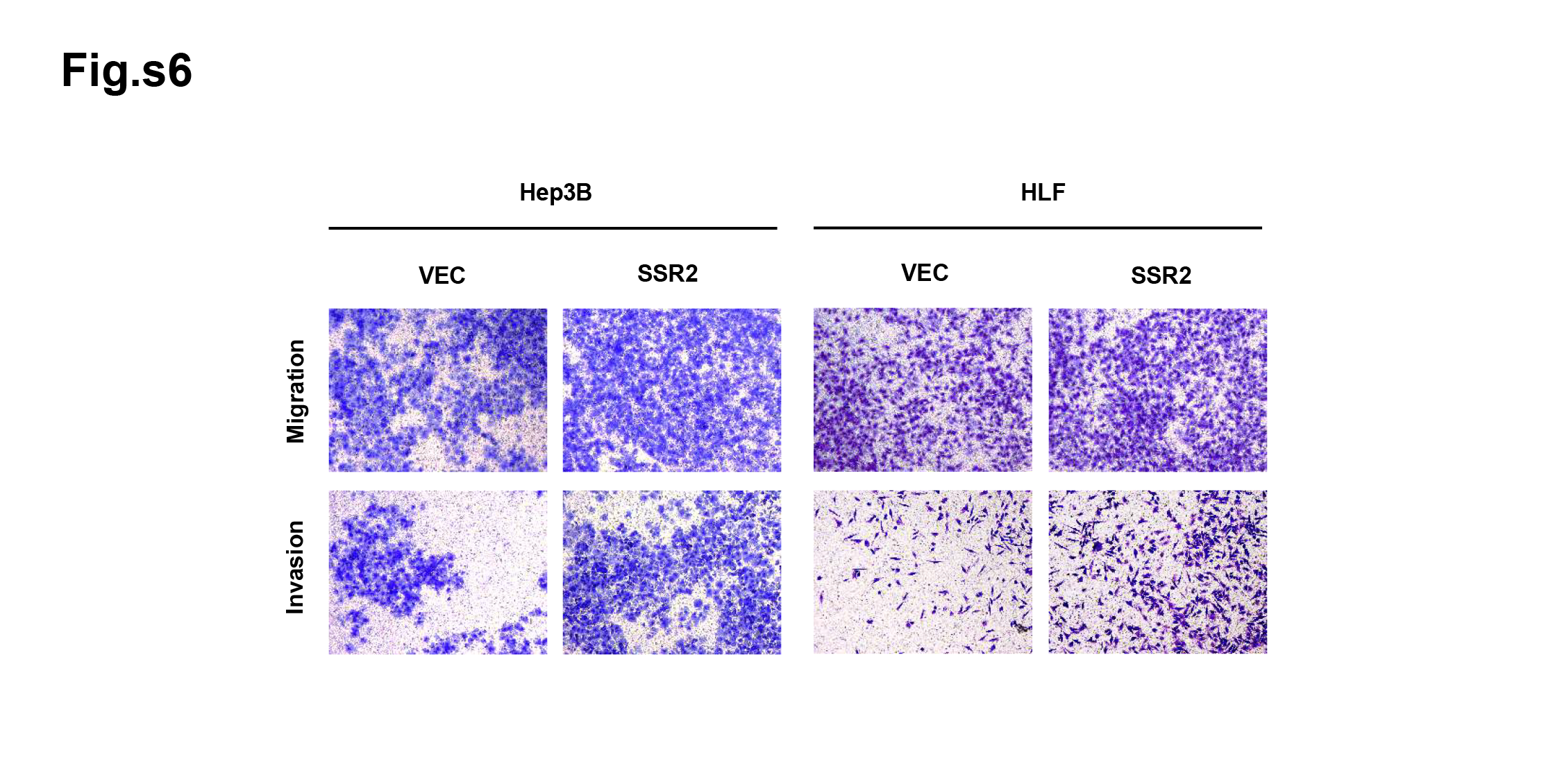

Supplement: Supplementary file 5 — Additional file 5: Supplementary Figure 6. SSR2 promotes migration and invasion of HCC cells in vitro. [file 13046_2021_1838_MOESM5_ESM.tif]

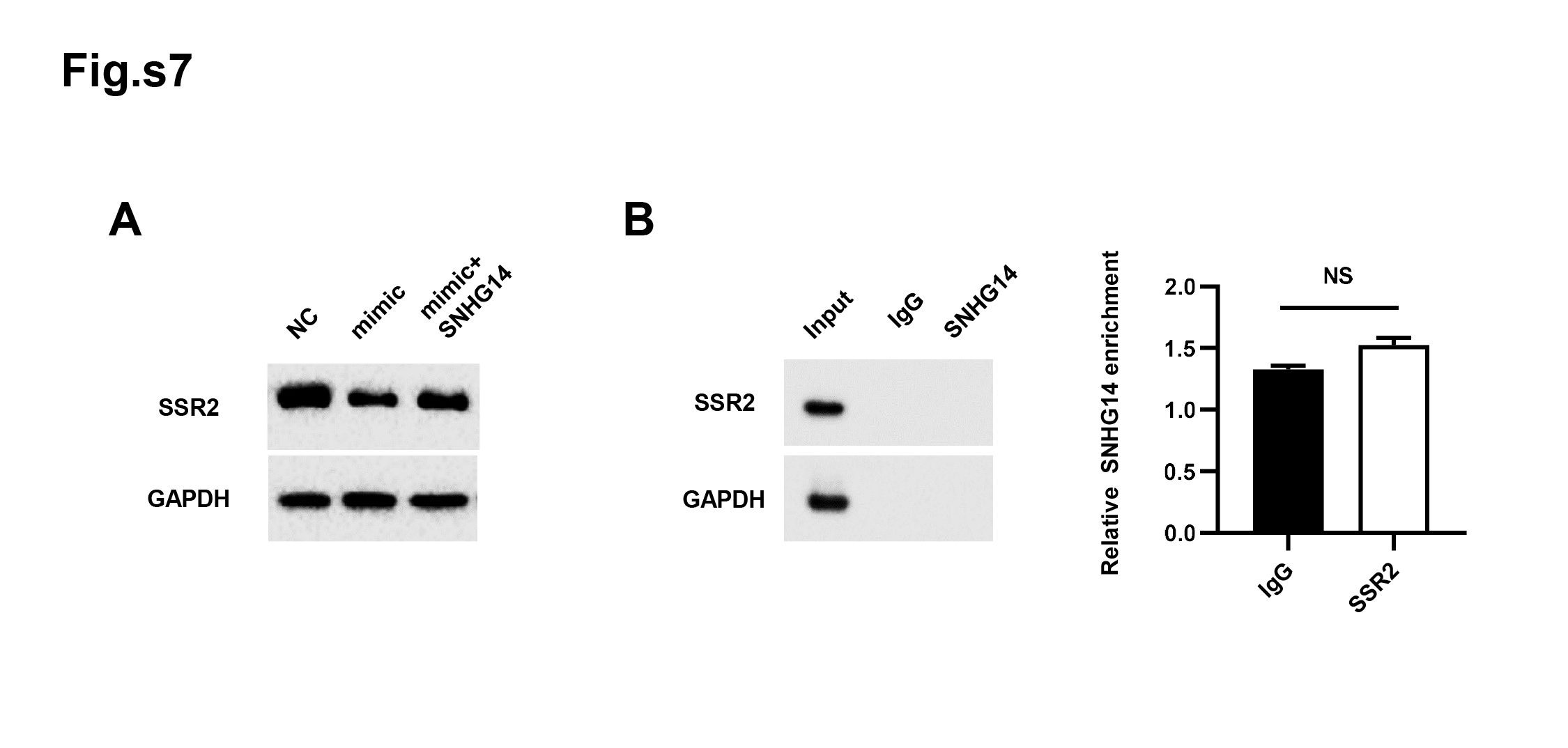

Supplement: Supplementary file 6 — Additional file 6: Supplementary Figure 7. SNHG14 regulates SSR2 to promote HCC progression dependent on miR-876-5p. (A) SSR2 protein levels in HLF after transfecting with miR-876-5p mimic and with or without SNHG14. (B) RIP assay was performed to identify the combination between SNHG14 and SSR2. [file 13046_2021_1838_MOESM6_ESM.tif]
