# Supplementary material for: Long noncoding RNA SNHG14 promotes hepatocellular carcinoma progression by regulating miR-876-5p/SSR2 axis
Source: J Exp Clin Cancer Res. 2021 Jan 23;40:36. doi: 10.1186/s13046-021-01838-5 (PMC7824933; doi:10.1186/s13046-021-01838-5)
Supplement: Supplementary file 7 — Additional file 7: Supplementary Table 1. Clinicopathologic characteristics of patients with hepatocellular carcinoma. [file 13046_2021_1838_MOESM7_ESM.docx]

**Supplementary Table 1.**

Clinicopathologic characteristics of patients with hepatocellular carcinoma.

| **Clinicopathological**  **variables** | **n=66**  **Number** | **Percentage** |
| --- | --- | --- |
| **Gender**  Male  Female | 56  10 | 0.85  0.15 |
| **Age**  ≤50  > 50 | 35  31 | 0.53  0.47 |
| **AFP (ug/L)**  ≤20  > 20 | 23  43 | 0.35  0.65 |
| **GGT(u/l)**  ≤54  > 54 | 42  24 | 0.64  0.36 |
| **ALT(ng/ml)**  ≤75  >75 | 55  11 | 0.83  0.17 |
| **HBV**  Negative  Positive | 9  57 | 0.14  0.86 |
| **Cirrhosis**  No  Yes | 21  45 | 0.32  0.68 |
| **Tumor size (cm)**  ≤5  >5 | 38  28 | 0.58  0.42 |
| **Tumor encapsulation**  Complete  None | 31  35 | 0.47  0.53 |
| **Tumor number**  Single  Multiple | 37  29 | 0.56  0.44 |
| **Differentiation**  I- II  III-IV | 37  29 | 0.56  0.44 |
| **BCLC stage**  0+A  B+C | 49  17 | 0.74  0.28 |
