# Supplementary material for: Long noncoding RNA SNHG14 promotes hepatocellular carcinoma progression by regulating miR-876-5p/SSR2 axis
Source: J Exp Clin Cancer Res. 2021 Jan 23;40:36. doi: 10.1186/s13046-021-01838-5 (PMC7824933; doi:10.1186/s13046-021-01838-5)
Supplement: Supplementary file 8 — Additional file 8: Supplementary Table 2. Correlation between relative SNHG14 expression and clinicopathologic characeristics in HCC patients (n = 66). [file 13046_2021_1838_MOESM8_ESM.docx]

**Supplementary Table 2.**

Correlation between relative SNHG14 expression and clinicopathologic characteristics in HCC patients (n = 66).

| **Clinicopathological**  **variables** | **Relative SNHG14 Expression** | | **P value** |
| --- | --- | --- | --- |
|  | **Low** | **High** |  |
| **Gender**  Male  Female | 24  7 | 32  3 | 0.107 |
| **Age**  ≤50  > 50 | 14  17 | 21  14 | 0.323 |
| **AFP (ug/L)**  ≤20  > 20 | 12  19 | 11  24 | 0.609 |
| **GGT(u/l)**  ≤54  > 54 | 18  13 | 24  11 | 0.446 |
| **ALT(ng/ml)**  ≤75  >75 | 25  6 | 30  5 | 0.743 |
| **HBV**  Negative  Positive | 1  30 | 8  27 | **0.021** |
| **Cirrhosis**  No  Yes | 9  22 | 12  23 | 0.792 |
| **Tumor size (cm)**  ≤5  >5 | 17  14 | 21  14 | 0.804 |
| **Tumor encapsulation**  Complete  None | 14  17 | 17  18 | 0.810 |
| **Tumor number**  Single  Multiple | 17  14 | 20  15 | 1.000 |
| **Differentiation**  I- II  III-IV | 15  16 | 22  13 | 0.321 |
| **BCLC stage**  0+A  B+C | 27  4 | 22  13 | **0.047** |
