# Supplementary material for: Long noncoding RNA SNHG14 promotes hepatocellular carcinoma progression by regulating miR-876-5p/SSR2 axis
Source: J Exp Clin Cancer Res. 2021 Jan 23;40:36. doi: 10.1186/s13046-021-01838-5 (PMC7824933; doi:10.1186/s13046-021-01838-5)
Supplement: Supplementary file 9 — Additional file 9: Supplementary Table 3. Correlation between relative has-miR-876-5p expression and clinicopathologic characeristics in HCC patients (n = 66). [file 13046_2021_1838_MOESM9_ESM.docx]

**Supplementary Table 3.**

Correlation between relative has-miR-876-5p expression and clinicopathologic characteristics in HCC patients (n = 66).

| **Clinicopathological**  **variables** | **Relative miR-876-5p Expression** | | **P value** |
| --- | --- | --- | --- |
|  | **Low** | **High** |  |
| **Gender**  Male  Female | 34  3 | 22  7 | 0.073 |
| **Age**  ≤50  > 50 | 22  15 | 13  16 | 0.175 |
| **AFP (ug/L)**  ≤20  > 20 | 15  22 | 8  21 | 0.202 |
| **GGT(u/l)**  ≤54  > 54 | 27  10 | 15  14 | 0.064 |
| **ALT(ng/ml)**  ≤75  >75 | 32  5 | 23  6 | 0.327 |
| **HBV**  Negative  Positive | 6  31 | 3  26 | 0.376 |
| **Cirrhosis**  No  Yes | 13  24 | 8  21 | 0.599 |
| **Tumor size (cm)**  ≤5  >5 | 19  18 | 19  10 | 0.183 |
| **Tumor encapsulation**  Complete  None | 16  21 | 15  14 | 0.331 |
| **Tumor number**  Single  Multiple | 20  17 | 17  12 | 0.452 |
| **Differentiation**  I- II  III-IV | 25  12 | 12  17 | **0.030** |
| **BCLC stage**  0+A  B+C | 24  13 | 25  4 | **0.044** |
